# Instruction Not Endorsement

**Published:** 1881-06

**Authors:** T. F. Pomeroy

**Affiliations:** Jersey City


### INSTRUCTION NOT ENDORSEMENT.

EDITOR HOMŒOPATHIC PHYSICIAN.—In reviewing, and much more in criticizing, a paper it is incumbent upon the critic, especially the editorial critic, that he shall *accurately* represent the views and statements of the one criticized. Your reply to my "Open Letter" headed "Endorsment" in the May number of your journal, in a marked manner violates this requirement and places me before your readers in an attitude that I have not assumed, one that my paper does not warrant and therefore one that I am not *required* to defend.

My "Open Letter" was strictly directed to the following proposition viz. "Is it not also a fatal error if the adherents of Hahnemann's strict inductive method endorse eclectic journals, such as publish eclectic papers, by writing truly homœopathic papers for them." It took in no other subject than that of writing truly homœopathic papers for eclectic journals. It did not consider adversely or otherwise the subject of "attending their society meetings" although it quoted Dr. Ad. Lippe's approval of so doing, so far at least as the American Institute of Homœopathy is concerned, which you affirm "has degenerated into eclecticism!" and is therefore no more nor no less than an eclectic association, in your opinion. I do not agree with you in that opinion; and the inference is a legitimate one that Dr. Lippe does not; it is much more than eclectic, it is a homœopathic institution. A "teacher" does not usually "award certificates" until after the period of "*instruction*" is completed, or is supposed to be sufficiently completed to warrant it, and therefore he can not "endorse" until then; so also may not the "homœopath endorse the eclectic" until he has completed the period of instruction; thus it is that teaching him is one office, endorsing him is quite another, and one does not necessarily imply the other, as you have assumed. My use of the term "*endorsement*" with reference to the Institute was manifestly borrowed from Dr. Lippe, and from his suggestion that writing papers for eclectic journals was an endorsement of them, which I have shown that it is not, no more than is a membership in the Institute an unlimited endorsement of it, and much less of the adverse opinion of one of its members "that any kind of disease could be cured with the 30th attenuation of medicine" Dr. Mc'Manus to the contrary notwithstanding, as you have quoted.

*The Institute* is not on record as having dissented from the *therapeutic views* of Dr. Mc'Manus and Dr. Berridge, this was the record of individual members of it only, and therefore you do it, and a large proportion of its members a great injustice in attempting to make it appear otherwise. So also you do both a great injustice in asserting, and that without the least evidence, that the "members [of the Institute] do not want to be taught, they feel no need of instruction!" The evidence and all the proofs point in quite a different direction, although there are undeniably those of its membership who would spurn, with the greatest scorn, such teaching as you and I would vouchsafe, but these members do not constitute the Institute.

It seems quite unnecessary, and a work of supererogation for you to attempt to frighten any old stager in pure homœopathic therapeutics with the fear that he may by any possibility be guilty of introducing some unkempt (homœopathically) eclectic. with his big doses of morphine or quinine, to the bedside of his patients; he is too cute for that, and the young stagers have cut their eye teeth long ago. Your apprehensions and cautions in this regard are altogether superflous. Such impositions are hardly possible under "the strict inductive method of Hahnemann" nor upon those who follow them.

T. F. Pomeroy.

Jersey City, May, 1881.

### REPLY.

Dr. Pomeroy claims that we have misrepresented him; we think not, but if we have done so we beg pardon as we do not wish to misrepresent any one, much less Dr. Pomeroy. We did not mean to assert in our article on "Endorsement" that all members of the American Institute of Homœopathy were eclectics, or inclined to eclecticism, but we do assert that the majority—and the majority rules—are so inclined. There are many very able and pure Hahnemannians in that Institute, but they are sadly in the minority. This Dr. Pomeroy acknowledges in both of his letters.

We would not be placed on record as opposing instruction. We desire that all, allopath and eclectic, shall learn of, and follow Hahnemann. If any of these want pure instruction can not they seek it? Read pure homœopathic journals; study Hahnemann's "Organon," etc.? When a man seeks allopathic or eclectic literature, it is reasonable to suppose that he does so because he would know of the virtues of allopathy or eclecticism; he does not seek homœopathy in allopathic journals, why then should he seek for it in the eclectic? Let allopathy, eclecticism, and homœopathy each have their societies and journals, then the student can choose which he desires. Let us have no mixtures; truth and error can not be mixed.—Editor.

